# Revisiting the Role of Ethylene and N-End Rule Pathway on Chilling-Induced Dormancy Release in Arabidopsis Seeds

**DOI:** 10.3390/ijms19113577

**Published:** 2018-11-13

**Authors:** Xu Wang, Zhazira Yesbergenova-Cuny, Catherine Biniek, Christophe Bailly, Hayat El-Maarouf-Bouteau, Françoise Corbineau

**Affiliations:** CNRS, Laboratoire de Biologie du Développement, Sorbonne Université, Boîte 24, 4 Place Jussieu, 75005 Paris, France; xu.wang@upmc.fr (X.W.); cuny@mail.ru (Z.Y.-C.); biniekcatherine@gmail.com (C.B.); christophe.bailly@upmc.fr (C.B.); hayat.bouteau@upmc.fr (H.E.-M.-B.)

**Keywords:** *Arabidopsis thaliana*, seed dormancy, cold, ethylene, gibberellins, N-end rule pathway

## Abstract

Dormant Arabidopsis (*Arabidopsis thaliana*) seeds do not germinate easily at temperatures higher than 10–15 °C. Using mutants affected in ethylene signaling (*etr1*, *ein2* and *ein4*) and in the N-end-rule pathway of the proteolysis (*prt6* and *ate1-ate2*) we have investigated the effects of cold and ethylene on dormancy alleviation. Ethylene (10–100 ppm) and 2–4 days chilling (4 °C) strongly stimulate the germination of wild type (Col-0) seeds at 25 °C. Two to four days of chilling promote the germination at 25 °C of all the mutants suggesting that release of dormancy by cold did not require ethylene and did not require the N-end-rule pathway. One mutant (*etr1*) that did not respond to ethylene did not respond to GA_3_ either. Mutants affected in the N-end rule (*prt6* and *ate1-ate2*) did not respond to ethylene indicating that also this pathway is required for dormancy alleviation by ethylene; they germinated after chilling and in the presence of GA_3_. Cold can activate the ethylene signaling pathway since it induced an accumulation of *ETR1*, EINI4, and *EIN2* transcripts, the expression of which was not affected by ethylene and GA_3_. Both cold followed by 10 h at 25 °C and ethylene downregulated the expression of *PRT6*, ATE1, ATE2, and of *ABI5* involved in ABA signaling as compared to dormant seeds incubated at 25 °C. In opposite, the expression of *RGA*, GAI, and *RGL2* encoding three DELLAs was induced at 4 °C but downregulated in the presence of ethylene.

## 1. Introduction

Seed dormancy is an important component of plant fitness that results in a delay of germination [1]. It corresponds to the inability to germinate of seeds even placed under apparently favorable conditions [1,2]. Dormancy and germination are characterized by a different balance between abscisic acid (ABA) and gibberellins (GAs) levels and metabolism [3,4,5]. ABA mainly plays a key role in the induction or maintenance of dormancy, while GAs are involved in germination and release of dormancy [6,7]. DELLAs negatively regulate the effects of GAs [8] and among them RGL2 (RGA-like 2) has been demonstrated as a regulator of dormancy in Arabidopsis by controlling ABA synthesis and activation of ABI5 (ABA insensitive 5) [9]. In addition, other hormones, ethylene (C_2_H_4_) in particular, participates in the control of seed germination [10,11,12]. Exogenous ethylene can break primary and secondary dormancies or stimulate germination of non-dormant seeds incubated in unfavorable conditions [10,11,12].

Treatment of seeds by chilling (2 to 5 °C) also promotes seed germination in many species [1,13] including Arabidopsis [14,15]. The stimulatory effect of cold is associated with an enhancement in seed sensitivity to GAs and an induction of GAs biosynthesis through changes in transcript abundance involved in GAs biosynthesis (*gibberellin 3-oxidase 1* = *GA3OX1*, *gibberellin 20 oxidase 1* = *GA20OX1*, GA20OX2) and GA degradation (*gibberellin 2 oxidase 2* = *GA2OX2*), and a decrease in ABA through induction of *abscisic acid 8*′*-hydroxylase 2* (*CYP707A2*) [7,9,13,16,17]. Cold treatment also affects ABA and GA_S_ signaling pathway associated proteins; for example, ABI5 and RGL2 decrease with loss of dormancy in Norway maple seeds [18]. Breaking of dormancy in Arabidopsis seeds by chilling is also associated with a decrease in expression of the *ACO* (*1-aminocyclopropane-1-carboxylic acid oxidase*) gene and a transient increase in *ACS* (*1-aminocyclopropane-1-carboxylic acid synthase*) expression [19]. In addition, low temperatures inhibit the ethylene production, but have a differential inhibitory effect on the activity of ACO and ACS, ACO being more inhibited than ACS, resulting in an accumulation of ACC in the tissues, and then a burst of ethylene production after transfer to 20–25 °C [20].

Identification of ethylene-insensitive and constitutive-response mutants by “triple response” in Arabidopsis, and the subsequent genetic study of these mutants have identified the key components of the ethylene biosynthesis and signaling pathways [21,22]. The ethylene-signaling cascade starts with ethylene binding to its receptors, which include ETR1 (ethylene receptor 1), ETR2, ERS1 (ethylene response sensor 1), ERS2, and EIN4 (ethylene insensitive 4). In the absence of C_2_H_4_, the receptors activate CTR1 (constitutive response 1), and CTR1 inactivates EIN2, by directly phosphorylating its C-terminal end. The level of EIN2 is negatively regulated by the F-box proteins ETP1/2 (EIN2 targeting protein 1/2) through proteasome. In the nucleus, the transcription factors EIN3/EIL1 (EIN3 like 1) are degraded by the other 2 F-box proteins, EBF1/2 (EIN3 binding F-box 1/2) through the proteasome. In the absence of EIN3/EIL1, transcription of the ethylene response genes is shut off. In the presence of ethylene, the receptors bind the hormone and become inactivated, which in turn, switches off CTR1. This inactivation prevents the phosphorylation of EIN2. The C-terminal end of EIN2 is cleaved off by an unknown mechanism and moves to the nucleus where it stabilizes EIN3/EIL1 and induces degradation of EBF1/2. The transcription factors EIN3/EIL1 dimerize and activate the expression of ethylene target genes, including ethylene response factors (ERFs), which are one of the largest subfamilies of the apetala2 (AP2)/ERF transcription factor family. Many proteins in the ERF family were identified and implicated in various functions in cellular processes, such as hormonal signal transduction, response to biotic, and abiotic stresses and in developmental processes in various plant species [23]. Ethylene breaks seed dormancy by decreasing seed sensitivity to ABA, and by inhibiting ABA signaling [10,11,12,24,25]. Numerous data demonstrates that exogenous ethylene promotes seed germination by stimulating GAs biosynthesis [12,26,27]. In Arabidopsis, it was also reported that repression of growth by ethylene is regulated by its effects on the DELLA proteins [28].

The ubiquitin-proteasome system (UPS) degrades the proteins by targeting specific signals, and contributes to numerous plant hormone signaling, in particular that of ABA, GAs, and ethylene [29,30,31]. As a part of the ubiquitin system, the N-end rule pathway regulates the in vivo half-life of the proteins depending on their N-terminal residue [32,33]. The essential components of N-end rule, which recognize the destabilized N-terminal residues (N-degrons), called N-recognins, belong to specific E3 ligase of UPS [33]. In general, tertiary destabilizing residues (Asn, Gln and Cys) are first modified, either enzymatically (by deamidation of Asn or Gln) or chemically (by oxidation of Cys), to generate a secondary destabilizing residue (Asp, Glu and oxidized Cys (C*)), respectively. Secondary destabilizing residues arginylated by Arg-tRNA protein transferase (ATE) generate destabilizing residue (Arg) [33,34]. Primary destabilizing residues fall into two categories, type 1 (Arg, Lys and His) and type 2 (Phe, Tyr and Trp) which are recognized by E3 specific ligases (N-recognins), PROTEOLYSIS 6 (PRT6) and PROTEOLYSIS 1 (PRT1), respectively [33,34]. In response to GAs, DELLA proteins are targeted for degradation via the ubiquitin-26S proteasome pathway [30,31,35,36,37,38]. The physiological effect of ABA, including seed germination is controlled by protein degradation [31]. ABA biosynthesis and signaling including the level of ABI5 are regulated by the UPS activity [31,39,40], and the N-end rule plays a role in seed sensitivity to ABA [41]. At least, stability of the aminocyclopropane-1-carboxylic acid synthase (ACS4, ACS5 and ACS9) enzyme involved in ethylene biosynthesis is regulated by the ubiquitin-dependent degradation [42,43], and proteasomal degradation of EIN3 and EIL1 transcription factors depends on ethylene concentration [44]. In addition, recently, ERFs from group VII which are characterized by Met-Cys at N-terminal have been identified as substrates of the N-end rule [45].

The purpose of the present work was (1) to determine the effects of cold, ethylene, and exogenous GA_3_ on the germination of dormant Arabidopsis seeds; (2) to investigate whether the responsiveness to chilling and GA_3_ involved C_2_H_4_ signaling using various mutants of this pathway; (3) to determine the role of the N-end rule on the responsiveness to ethylene, chilling, and GA_3_; and (4) to investigate the effects of chilling and ethylene that both alleviate seed dormancy on the expression of genes involved in the ethylene signaling pathway (*ETR1*, EIN4, and *EIN2*), N-end rule pathway (*PRT6*, ATE1, ATE2), and of *ABI5*, gene encoding ABI5, regulator of ABA action, and three genes encoding DELLAs (*gibberellin insensitive* = *GAI*, *repressor of ga1-3* = *RGA* and *RGA-like 2* = *RGL2*) which regulate the GAs signaling.

## 2. Results

### 2.1. Effects of Cold, Ethylene and GA_3_ on Germination of Dormant Seeds

**Figure 1:**
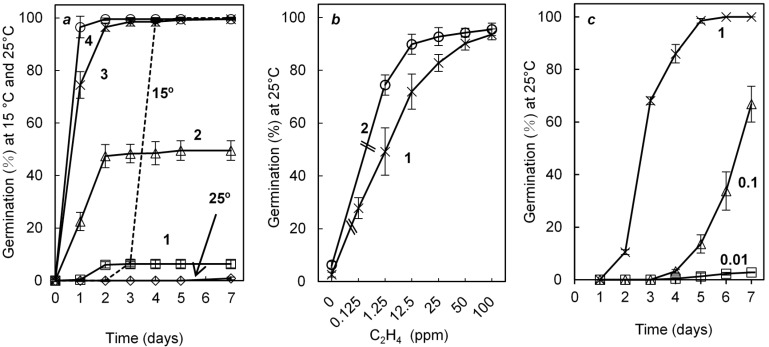
Effects of temperature (**a**), ethylene (**b**) and GA_3_ (**c**) on the germination of dormant Col-0 seeds. (**a**) germination at 15 °C (---) and 25 °C (indicated by arrow, open diamonds), and at 25 °C after one (1), two (2), three (3), and four (4) days of incubation at 4 °C; (**b**) effects of ethylene concentration on the germination percentages obtained after seven days at 25 °C with seeds placed directly at 25 °C in the presence of C_2_H_4_ (1) or after one day of incubation at 4 °C (2); (**c**) effects of GA_3_ concentration (mM) on the germination percentage obtained at 25 °C. Seeds harvested in 2014. Means of 3 replicates ± SD.

Dormant Arabidopsis (Col-0) seeds were able to easily germinate at 15 °C but no germination occurred at 25 °C (Figure 1a). This dormancy was progressively broken by an incubation at 4 °C (Figure 1a). After one, two, three, and four days at 4 °C, 6.3, 49.5, and 99.5% of the seed population became able to germinate at 25 °C within two days (Figure 1a). Moreover, 0.125 ppm exogenous ethylene improved the germination at 25 °C. This stimulatory effect increased with increasing ethylene concentration, around 50% and 100% of the seed population being able to germinate at 25 °C within seven days in the presence of ethylene at 1.25 ppm and 50–100 ppm, respectively (Figure 1b). Incubation of dormant seeds for one day at 4 °C did not strongly improve the germination in air, but had an additive effect with ethylene; around 75% of the seed population became able to germinate in the presence of 1.25 ppm ethylene against 50% for the control non-chilled seeds (Figure 1b). In contrast, 1-methylcyclopropene (1-MCP) 1000 ppb, an inhibitor of ethylene action, resulted in an inhibition of germination at 15 °C and a suppression of the improving effect of ethylene at 25 °C (Table S1).

GA_3_ at high concentration (0.1 and 1 mM) improved the germination of dormant seeds placed at 25 °C (Figure 1c). All the seed population germinated within five days at 25 °C in the presence of GA_3_ 1 mM, when only 2.8% and 66.8% of the seed population germinated within seven days in the presence of GA_3_ 0.01 and 0.1 mM, respectively (Figure 1c). In addition, the stimulatory effect of ethylene (12.5 and 50 ppm) was reduced in the presence of ABA, and suppressed in the presence of ABA 10 μM (Figure S1).

### 2.2. Involvement of Ethylene Signaling Pathway and of the N-End Rule Pathway on the Responsiveness of Dormant Seeds to Cold, Ethylene and GA_3_

In order to determine the involvement of ethylene in the regulation of dormancy, we have used various mutants (*etr1*, *ein2*, and *ein4*) affected in the ethylene signaling pathway at the level of the receptor (ETR1 and EIN4), and of EIN2 which play a central role in the responsiveness to this hormone [12,46,47]. Seeds of Col-0 and all the mutants studied did not germinate at 25 °C, but easily germinate at 15 °C except *etr1* and *ein2* mutants (Table 1). The same behavior was observed with seeds harvested in 2016; the germination of *etr1* and *ein2* seeds was lower at 15 °C than that of Col-0, and neither of the mutants replied to exogenous ethylene (Table S2).

**Figure 2:**
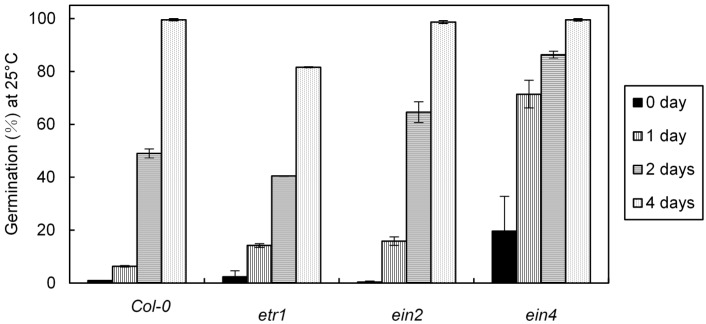
Effects of seed stratification at 4 °C for one, two, three, and four days on the germination percentages obtained after seven days at 25 °C with various genotypes affected in the ethylene signaling pathway (*etr1*, *ein2* and *ein4*). Means of three replicates ± SD.

*Etr1* and *ein2* were almost totally insensitive to C_2_H_4_ (Table 1 and Table S2), but three to four days of chilling at 4 °C promoted germination at 25 °C of all mutants including the ethylene insensitive ones (*etr1*, *ein2*) (Table 1, Figure 2) suggesting that cold could act directly on dormancy by bypassing the ethylene pathway even though *etr1* remained the less sensitive to stratification (Figure 2).

GA_3_ at high concentration (1 mM) promoted the germination of dormant seeds in Col-0 (Figure 1c). Among the mutants studied, *ein2* seeds were responsive to GA_3_ to a high extent as germination reached around 40% and 80% in the presence of GA_3_ 0.1 mM (data not shown) and 1 mM (Table 1), respectively. In contrast, *etr1* did not respond to GA_3_ (Table 1), and it was also more sensitive to paclobutrazol (1 µM) at 15 °C (Table 1).

**Figure 3:**
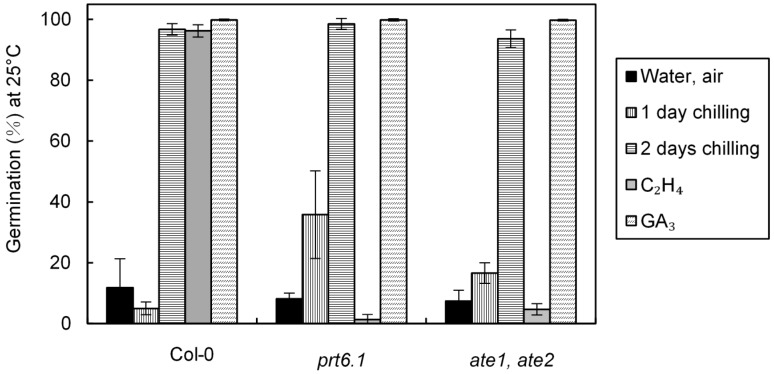
Germination percentages obtained after seven days at 25 °C with Col-0 and the N-end rule mutants (*prt6-1* and *ate1-ate2)* seeds placed directly at 25 °C on water without ethylene (control) or in the presence of ethylene 100 ppm, at 25 °C in the presence of GA_3_ 1 mM and at 25 °C after 1 and 2 days of incubation at 4 °C. Means of three replicates ± SD. Seeds harvested in 2016.

Ethylene did not break the dormancy of seeds from *prt6* mutant and *ate1-ate2* double mutant suggesting that the N-end rule is involved in seed responsiveness to ethylene (Figure 3). Like the mutants affected in the ethylene signaling pathway (*etr1* and *ein2*) (Table 1), 2–4 days of incubation at 4 °C promoted the germination of almost all the seed population from the mutants affected in the N-end rule pathway (Figure 3). Unlike *etr1* (Table 1), the germination of *prt6* and *ate1-ate2* mutants was promoted by GA_3_ (1 mM) (Figure 3).

### 2.3. Effect of Cold, Ethylene and GA_3_ Treatments on the Relative Expression of ETR1, EIN4, and EIN2 Involved in Ethylene Signaling Pathway

**Figure 4:**
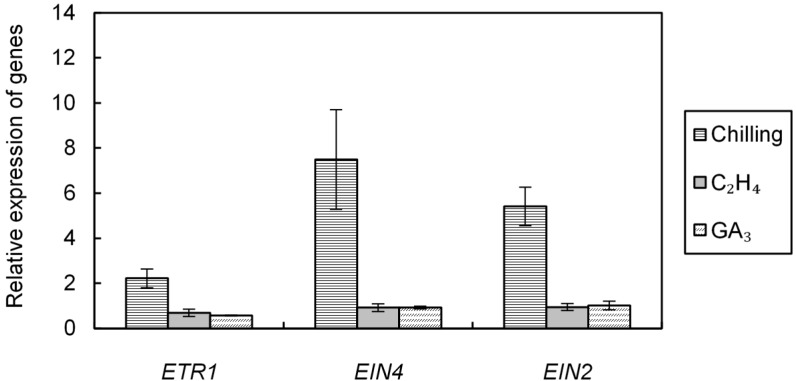
Relative expression of *ETR1*, EIN4, and *EIN2* in seeds (Col-0) incubated for 24 h at 4 °C, at 25 °C in the presence of ethylene or GA_3_ 1 mM. Control seeds were incubated for 24 h at 25 °C on water, an arbitrary value of 1 was assigned to control seeds incubated for 24 h at 25 °C on water. Means of three replicates ± SD. Seeds harvested in 2014.

Figure 4 shows the effects of incubation of wild type seeds (Col-0) for 24 h at 4 °C, at 25 °C in the presence of ethylene or GA_3_ on the relative expression of *ETR1*, *EIN4*, and *EIN2* calculated as compared to control seeds imbibed for 24 h on water without either ethylene or cold. One day at 4 °C strongly induced the relative expression of the gene involved in ethylene perception (*ETR1*, *EIN4*) and the major signaling elements gene (*EIN2*) (Figure 4) indicating the involvement of ethylene in the cold response. After 24 h treatment at 4 °C, the transcript abundance was 2.21, 5.40, and 7.50 times of the transcript abundance measured in the control seeds incubated at 25 °C, for *ETR1*, *EIN2*, and *EIN4*, respectively. In contrast, 24 h-incubation in the presence of either ethylene or GA_3_ (Figure 4) did not significantly induced changes in the relative expression of the three genes.

Seed incubation for 16 h increased both gene expressions as compared to the dry seeds (Table 2). Longer incubation (30 and 48 h) at 25 °C in the presence of ethylene results then in a decrease in *ERT1* and *EIN2* expression as compared to seeds incubated without ethylene (Table 2).

### 2.4. Effect of Cold and Ethylene Treatments on the Relative Expression of PRT6, ATE1, and ATE2 Involved in the N-End Rule

**Figure 5:**
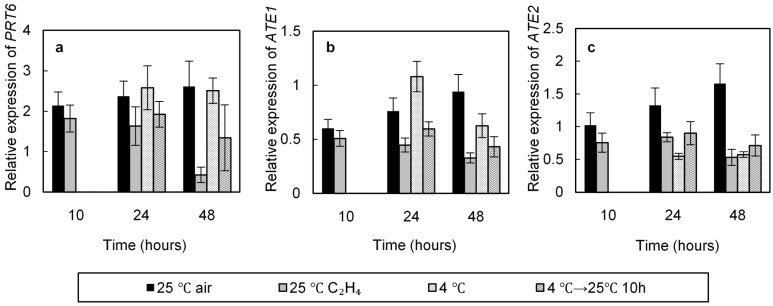
Relative expression of *PRT6* (**a**), *ATE1* (**b**), and *ATE2* (**c**) in seeds (Col-0) incubated at 25 °C or at 4 °C on water, at 25 °C for 10 h after stratification at 4 °C, and at 25 °C in the presence of ethylene 100 ppm. An arbitrary value of 1 was assigned to dry seeds. Means of three replicates ± SD. Seeds harvested in 2016.

Incubation at 25 °C, a temperature at which wild type seeds (Col-0) did not germinate, resulted in an increase in *PRT6* and *ATE2* relative expression which was 2.62 and 1.66 times higher than that in the dry seeds after 48 h of incubation (Figure 5a,c), but did not result in significant changes in *ate1* transcript relative abundance (Figure 5b). Seed incubation at 4 °C, a temperature which progressively alleviated dormancy was also associated with an increase in *PRT6* similar to that observed at 25 °C (Figure 5a) and a decrease in *ATE1* and *ATE2* expression (Figure 5b,c). However, transfer of seeds from 4 °C to 25 °C for 10 h resulted in a strong decrease in *PRT6* relative expression (Figure 5a). This down-regulation of *PRT6* and *ATE1* from 24 h (Figure 5a,b) was associated with breaking of dormancy or the start of the germination process.

Seed incubation at 25 °C in the presence of ethylene (100 ppm) resulted in a significant decrease in *PRT6*, ATE1, and *ATE2* transcript levels for 24 h of imbibition as compared with seeds placed at 25 °C without ethylene (Figure 5). After 48 h in the presence of ethylene the transcript abundance of *PRT6*, ATE1, and *ATE2* corresponded to around 0.43, 0.33, and 0.53 of the initial value, respectively, when *PRT6* relative expression reached 2.60 and *ATE1* and *ATE2* remained unchanged or less than double (0.94 and 1.66, respectively) for seeds incubated at 25 °C without ethylene (Figure 5).

### 2.5. Effect of Cold and Ethylene Treatments on the Relative Expression of ABI5, RGA, GAI, and RGL2

ABA and GAs being involved in the germination of dormant seeds, we have compared the effects of cold and ethylene on the expression of *ABI5*, involved in ABA signaling, in particular ABA inactivation, and *RGA*, GAI, and *RGL2* encoding three DELLAs involved in the regulation of GAs efficiency.

**Figure 6:**
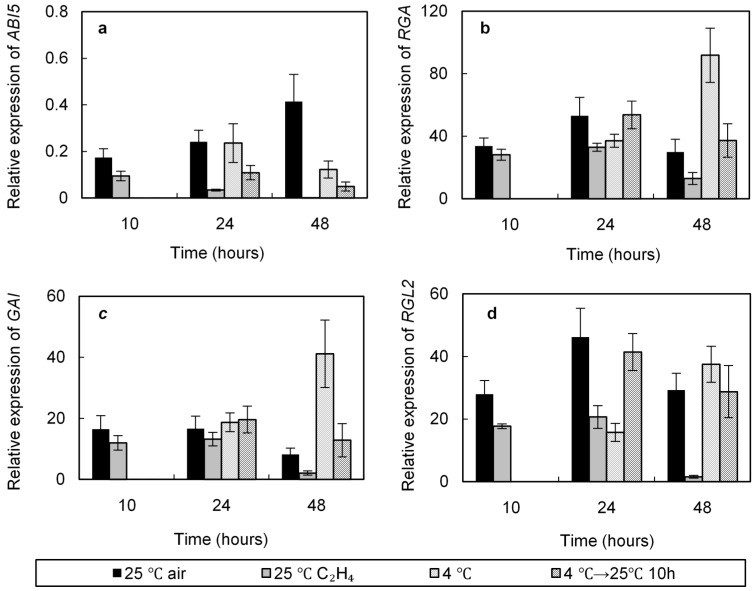
Relative expression of *ABI5* (**a**), *RGA* (**b**), *GAI* (**c**), and *RGL2* (**d**) in seeds (Col-0) incubated at 25 °C or at 4 °C on water, at 25 °C for 10 h after stratification at 4 °C and at 25 °C in the presence of ethylene 100 ppm. An arbitrary value of 1 was assigned to dry seeds. Means of three replicates ± SD. Seeds harvested in 2016.

Seed imbibed at either 25 or 4 °C resulted in a decrease in *ABI5* relative expression, this phenomenon being more important at 4 °C than that at 25 °C (Figure 6a). For example, after 48 h incubation the *ABI5* transcript relative abundance was 0.12 and 0.41 of its initial value, at 4 and 25 °C, respectively. Seed transferred from 4 °C to 25 °C resulted in an additional reduction of the transcript level. Ethylene applied at 25 °C almost completely suppressed the expression of *ABI5* after 24 h of incubation (Figure 6a).

Expression of the genes encoding the three DELLAs was induced during seed incubation at 4 and 25 °C, but this increase depended on the temperature (Figure 6b–d). Except for that of *RGL2* (Figure 6d), the relative expression of *RGA* (Figure 6b) and *GAI* (Figure 6c) was higher at 4 °C than that at 25 °C at 48 h, and transfer of seeds from 4 to 25 °C resulted in a decrease in the transcript relative abundance for the three *DELLA* genes. Application of ethylene at 25 °C for at least 24 h resulted in a decrease in the gene relative expression for the three *DELLAs* (Figure 6b–d). After 48 h of seed incubation in the presence of ethylene, the relative expression of *RGA*, GAI, and *RGL2* respectively represented 0.43, 0.26, and 0.05 of that calculated in the control seeds placed at 25 °C without ethylene.

## 3. Discussion and Conclusions

At harvest, Arabidopsis seeds easily germinate at low temperatures (10–15 °C), but their germination become progressively impossible with increasing temperature and does not occur at 25 °C (Figure 1a). Two days of incubation at 4 °C was enough to alleviate dormancy (Figure 1a) whereas removal of dormancy requires more than 2–8 weeks of seed stratification in other species [48]. The involvement of ABA and GAs, and the hormonal balance between both hormones in the regulation of dormancy in response to chilling is well documented [3,4,6,49]. Breaking of dormancy by cold treatment is associated with a decrease in ABA resulting in induction of *CYP707A2* and in ABA sensitivity, and an increase in GAs biosynthesis through changes in GA3OX1 and *GA20OX* expression [13,16,17,50,51]. Our results demonstrate that cold treatment breaks dormancy of all mutants affected in ethylene signaling, however *etr1* seeds have the lowest responsiveness to chilling suggesting that ethylene might be indirectly involved in dormancy (Figure 2, Table 1). Figure 4 also indicates that 24 h of cold stratification results in an up-regulation of *ETR1*, EIN4, and *EIN2* which can explain the increase in ethylene sensitivity after stratification of Arabidopsis (Figure 1b) and apple embryos [52]. Opposingly, incubation of seeds in the presence of GA_3_ and ethylene does not affect the expression of these three genes (Figure 4).

Exogenous ethylene also stimulates the germination of dormant Arabidopsis seeds (Figure 1b) as in other numerous species [10,11,12]. This stimulatory effect is dose dependent, and Figure 1b shows that 50–100 ppm (µL L^−1^) is optimal to alleviate seed dormancy. However seed responsiveness to ethylene depends on the species and the depth of dormancy [12]. Our results demonstrate that one day chilling is associated with an increasing sensitivity to ethylene (Figure 1b) in agreement with the results obtained during breaking of dormancy by chilling in apple [52] and during dry storage in sunflower [53]. In agreement with the results published by Beaudoin et al. [24] and Siriwitayawan et al. [54] *etr1* as well as *ein2* are more dormant than wild type ones (Table 1 and Table S2) demonstrating that lower responsiveness to ethylene is associated with dormancy. Our results also demonstrate that an inhibitor of ethylene action (MCP) inhibits seed germination at 15 °C (Table S1). The regulation of germination by ethylene in relation with ABA and GAs is well documented [11,12]. Among the mutant studied, Table 1 shows that at 25 °C *etr1* does not response to GA_3_, even applied at high concentrations (1 mM) and that the germination at 15 °C is strongly inhibited in the presence of paclobutrazol (1 μM); these results are in agreement with a crosstalk between ethylene and GAs signaling pathway, ETR1 being a possible sharing component. Chiwocha et al. [55] also indicates that lack of ETR1 leads to changes in GAs biosynthesis and a lower sensitivity to GAs. Our results also indicate that the improving effect of chilling and exogenous ethylene on seed germination do not result in similar changes in expression of *RGA*, *GAI*, and *RGL2*, genes encoding three DELLAs. After 48 h, the transcript abundance remains higher at 4 °C than that at 25 °C, temperature at which germination does not occur when application of ethylene results in a fast decrease in *RGA*, GAI, and *RGL2* relative expression (Figure 6b–d). Alleviation of dormancy after two days at 4 °C is not associated with a strong decrease of the three gene expression; after transfer of seeds from 4 °C to 25 °C (i.e., in non-dormant seeds), *RGA* (Figure 6b), *GAI* (Figure 6c), and *RGL2* (Figure 6d) relative expressions are similar to those measured in dormant seeds maintained at 25 °C. Achard et al. [28,56] reported that ethylene action is mediated via its effects on DELLAs proteins. The decrease in *DELLAs* transcripts in the presence of ethylene observed in Figure 6 could result from a crosstalk between GAs and ethylene signaling pathway. ABA counteracts the effect of C_2_H_4_ (Figure S1) and Beaudoin et al. [24] reported a high sensitivity to ABA of seeds from the mutant affected in the ethylene signaling pathway. Among the mutants studied, *etr1* is the more sensitive to ABA (Table S2). In addition we demonstrate that exogenous ethylene strongly down-regulates the expression of *ABI5* and that this decrease in *ABI5* expression is more important in the presence of ethylene than that at 4 °C (Figure 6a). Beside the effects of chilling on ethylene signaling pathway (Figure 4) and ethylene sensitivity (Figure 1b), stratification at low temperatures can improve germination after seed transfer at 25 °C through an effect on ABA and GAs signaling pathways, transcript abundance of *ABI5*, and *DELLA_S_* being modified (Figure 6).

It is well accepted that E3 ligase activity plays an essential role in hormone signal transduction pathway [29,30,31,36,57]. For the first time, we demonstrate that the seed responsiveness to ethylene requires the N-end rule pathway, dormancy of seeds from *prt6* and *ate1-ate2* mutants being insensitive to ethylene (Figure 3). As an *ein2* mutant (Table 1), dormancy of *prt6* and *ate1-ate2* mutants can be alleviated by 2–4 days of cold stratification or application of GA_3_ (Figure 3). As previously published by Holman et al. [41] and Gibbs et al. [45], *prt6* and *ate1-ate2* seeds are more sensitive to ABA and more tolerant to hypoxia than the wild type. In addition, the ethylene response factors from group VII (RAP2.2, RAP2.3, RAP2.12, HRE1, and HRE2) have been identified as substrates of the N-end rule pathway [45,58,59]. Gibbs et al. [59] demonstrate that the effect of NO which alleviates Arabidopsis dormancy [11] requires a regulation through the expression of *ABI5* [60]. Figure 5 indicates that exogenous ethylene and 2 day-chilling followed by 10 h at 25 °C, conditions which allow germination, result in a decrease of the relative expression of *PRT6* (Figure 5a), *ATE1* (Figure 5b), and *ATE2* (Figure 5c), whereas seed incubation at 25 °C which does not allow germination, does not significantly affect the relative expression of *PRT6*, ATE1, and *ATE2*. These data suggest that down-regulation of the genes involved in the N-end rule is associated with the germination of Col-0. Using mutants and as demonstrated by Gibbs et al. [59,60] concerning the seed responsiveness to NO, we suggest that the insensitivity to ethylene probably results from a regulation of group VII ERF levels via the N-end rule activity and a crosstalk with ABA and GAs signaling.

In conclusion, the present work demonstrates that cold treatment and exogenous ethylene, which both alleviate seed dormancy, do not result in comparable changes in expression of genes involved in the ethylene signaling pathway, in ABA signaling (*ABI5*) and in genes encoding DELLAs (Figure 4, Figure 5 and Figure 6). We report for the first time the involvement of the N-end rule pathway in the responsiveness of seeds to ethylene. The aims of our further researches are to determine whether the ERF transcription factors have a role in the interaction between the N-end rule pathway, ABA, and GAs signaling pathways in the regulation of dormancy.

## 4. Materials and Methods

### 4.1. Seed Material

*Arabidopsis thaliana* seeds from Columbia-0 (Col-0) were used as the wild type of this study. The mutants affected in the ethylene signaling pathway and the N-end rule pathway were obtained from the Nottingham Arabidopsis Stock Center (NASC) or from individual researchers: *etr1* (NASC ID number N237); *ein4* (NASC ID number N8053) [61]; *ein2* (NASC ID number N3071) [62]; *prt6-1* (Sail-1278-H11); *ate1-ate2* (gifts from Dr. M. J. Holdsworth, Nottingham University) [41,45,59,63]. All mutants were in the Arabidopsis genetic background Columbia-0. Seeds were sown in a mixture of soil and vermiculite (9:1) and placed in a growth chamber at 21 °C under a photoperiod of 16 h light/8 h dark with a light intensity between 150 and 200 µmol·m^−2^·s^−1^. After 12–15 days, germinated seeds were transferred on soil/perlite/vermiculite (2:1:1) and plants were grown in the same conditions. Siliques were harvested at maturity and seeds were collected. Seeds freshly harvested in 2014, 2015, and 2016 with a moisture content at around 5–6% dry weight were stored at −30 °C in order to preserve their initial dormancy.

### 4.2. Germination Assays

Germination assays were performed in darkness in 9 cm Petri dishes (100 to 200 seeds per assay in 3 replicates) by placing seeds on a filter paper on the top of a layer of cotton wool moistened with deionized water, abscisic acid (ABA: 0.1, 1 or 10 µM), gibberellic acid (GA_3_: 0.01, 0.1 or 1 mM), paclobutrazol, and 1-methylecyclopropene (1-MCP: 1000 ppb). Seed stratification was carried out at 4 °C as the germination assays on water for 1 to 4 days.

Germination assays in the presence of gaseous ethylene were carried out in tightly closed 360 mL-containers in which was injected gaseous ethylene (5%) (Air Liquide, Paris, France) in order to obtain concentration from 0 to 100 μL L^−1^. 

A seed was considered to have germinated as soon as the radicle protruded through the seed coat. Germination counts were made every 24 h for 7 days, and the results presented are the means of the germination percentages obtained with 3 replicates ± SD.

### 4.3. RNA Extraction and Real-Time Quantitative RT–PCR

A 50 mg aliquot of seeds was ground in liquid nitrogen, and total RNA was extracted by a modified CTAB method as described by Chang et al. [64]. Total RNA (1 µg) was treated with DNase I (ThermoFisher, Waltham, MA, USA), reverse transcribed with Revertaid Reverse Transcriptase (ThermoFisher) in a 25 µL reaction volume and amplified with Mastercycler ep Realplex (Eppendorf, Hamburg, Germany) using 5 µL of 30-fold diluted cDNA solution. Primers were designed with primer3 software. They were obtained from Eurogentec (Angers, France) and the primer sequences are shown in Table S1. Real-time PCRs were performed with the Maxima™ SYBR Green qPCR Master Mix (ThermoFisher) and 0.23 µM of each primer in a 15 µL reaction. Cycle thresholds (Cts) were calculated using the Realplex 2.0 software (Eppendorf). For each plate and each gene, a standard curve made with dilutions of cDNA pools was used to calculate the reaction efficiencies, and the relative expression was calculated according to Hellemans et al. [65] with *UBQ5* (*Ubiquitin5*, AT3G62250) and *EMB1345* (*Embryo defective 1345*, AT2G26060), or with *CB5-E* (*Cytochrome B5 isoform E*, AT5G53560), *RHIP1* (*Chromosome associated kinesin*, AT4G26410) and *TIP41* (*Tip-like family Protein*, AT4G34270) as reference genes used by [66] and by [67,68], respectively. An arbitrary value of 1 was assigned to the Col-0 seeds imbibed during 24 h at 25 °C on water (Figure 4) or to the dry seeds (Figure 5 and Figure 6), which were used as control for normalization [69]. Results presented are the means ± SD of 3 or 4 biological replicates.

## Figures and Tables

**Table 1:** Germination percentages obtained after seven days at 15 and 25 °C, at 15 °C in the presence of the paclobutrazol (1 µM) and at 25 °C in the presence of ethylene 50 ppm, GA_3_ 1 mM and after four days of incubation at 4 °C. Means of 3 replicates ± SD. Seeds harvested in 2014.

Lines	Germination (%) after 7 Days at					
	15 °C	15 °C with 1 μM Paclobutrazol	25 °C	25 °C with 50 ppm C_2_H_4_	25 °C after 4 Days at 4 °C	25 °C with 1 mM GA_3_
Col-0	100	91.5 ± 5.4	0.9 ± 0.1	96.3 ± 2.2	99.5 ± 0.3	100
*etr1*	42.4 ± 3.4	2.5 ± 0.9	2.3 ± 2.1	7.5 ± 0.3	81.6 ± 2.5	23.8 ± 13.0
*ein4*	100	76.9 ± 0.8	19.6 ± 10.4	75.6 ± 3.8	99.5 ± 0.2	88.0 ± 2.3
*ein2*	86.4 ± 2.5	95.1 ± 0.8	0.3 ± 0.3	2.8 ± 2.8	98.6 ± 0.3	81.1 ± 9.1

**Table 2:** Relative expression of *ETR1* and EIN2 in seeds (Col-0) incubated for 16, 30, and 48 h at 25 °C in the absence or the presence of ethylene 100 ppm. Calculation was done as compared to dry seeds. Means of three replicates ± SD. Seeds harvested in 2016.

Conditions of Seed Incubation at 25 °C		Relative Expression (Dry Seeds) of	
Treatment	Duration (h)	*ETR1*	*EIN2*
Air	16 30 48	3.09 ± 0.55 1.97 ± 0.27 5.20 ± 1.72	5.66 ± 0.27 3.16 ± 0.46 9.41 ± 0.67
Ethylene (100 ppm)	16 30 48	2.64 ± 0.38 1.20 ± 0.19 0.63 ± 0.16	4.98 ± 1.05 1.55 ± 0.18 1.72 ± 0.18

## Supplementary Materials

The following are available online at http://www.mdpi.com/1422-0067/19/11/3577/s1.
