# Supplementary material for: Revisiting the Role of Ethylene and N-End Rule Pathway on Chilling-Induced Dormancy Release in Arabidopsis Seeds
Source: Int J Mol Sci. 2018 Nov 13;19(11):3577. doi: 10.3390/ijms19113577 (PMC6275081; doi:10.3390/ijms19113577)
Supplement: Supplementary file 1 [file ijms-19-03577-s001.pdf]

Supplementary Table S1

Table S1. Germination percentages obtained after 7 days with Col-0 seeds incubated at 15 and 25°C in darkness, in absence or presence of 1000 ppb MCP. Means of 3 replicates ± SD. Seeds harvested in 2014.

Atmosphere	Germination (%) after 7 days at	
	15°C	25°C
Air	91,5 ± 3.4	2.6 ± 0.9
C_2H_4 50 ppm	95.5 ± 2.5	90.2 ± 2.5
C_2H_4 50 ppm+MCP 1000 ppb	34.3 ± 4.3	5.9 ± 0.5

Supplementary Table S2

Table S2. Germination percentages obtained after 7 days at 15 and 25°C in darkness, at 25°C in the presence of ethylene 100 ppm and after 4 days of incubation at 4°C. Means of 3 replicates ± SD. Seeds harvested in 2016.

Lines	Germination (%) after 7 days at			
	15°C	25°C	25°C with 100 ppm C_2H_4	25°C after 4 days at 4°C
Col-0	98.4 ± 1.5	0	96.5 ± 1.5	100
etr1	12.7 ± 3.9	0	1.9 ± 1.4	91.4 ± 4.1
ein2	45.2 ± 8.3	0	1.7 ± 0.7	98.0 ± 1.9

Supplementary Table S3

Table S3. Germination percentages obtained after 7 days at 15 °C in darkness, at 15°C in the presence of ABA (0.1, 1 and 10 μM). Means of 3 replicates ± SD. Seeds harvested in 2015.

Lines	Germination (%) after 7 days at 15°C on			
	Water	ABA 0.1 μM	ABA 1 μM	ABA 10 μM
Col-0	94.1 ± 0.2	98.7 ± 0.5	66.1 ± 4.1	29.5 ± 1.7
etr1	23.1 ± 4.3	32.6 ± 3.7	17.7 ± 4.8	3.8 ± 0.6
ein4	91.3 ± 2.3	89.7 ± 3.3	50.6 ± 7.3	11.5 ± 3.8
ein2	75.3 ± 6.2	85.2 ± 5.2	44.0 ± 4.3	8.6 ± 1.7

Supplementary Table S4

Table S4. List and sequences of the primers used in real time RT-PCR experiments.

Gene	Locus ref.	Forward primer	Reverse primer
AIN1	AT1G54490	TACCTGTGTCCTGCCAAACA	GCGTCGGTTGCTATTGTCTT
ETR1	AT1G66340	CGCCTTCTTCTCCCGTAAA	CCACCACCATCTTGTTCCTC
EIN2	AT5G03280	CACCACTCTGCTTGTATCGTC	TCGTCTTCGTTCCTCCTTGT
EIN4	AT3G04580	TGAAACGAAGAAAAGCAGAGTG	GTAGAACCGAGACAACAACAACAA
GAI	AT1G14920	ATCTTAAAGCTATTCCCGGTGAC	GTATCTCCTCCGCCGCCTTG
RGA	AT2G01570	CCAATTCCAAGGTCGATTGTCC	CTCGTCGTCCATGTTACCTCC
RGL2	AT3G03450	AAAACCACTACCAGCTTCTCGTT	CAGCCATCTCAGAAGATCGAAC
ABI5	AT2G36270	GAGAATGCGCAGCTAAAACA	GTGGACAACTCGGGTTCCTC
PRT6	AT5G02310	CGTTGGTCAGAACAGGGACTTG	ATAGCTTGAGAGCCCAAATCGACT
ATE1	AT5G05700	TATAGAGAACCAAGCTCGCTGCC	TCAAATGGAACCCACTGGAAACG
ATE2	AT3G11240	CGACTTCGATACAAGGATATACCG	GAGTTCATACACCATTATCTCCGA
UBQ5	AT3G62250	CTTGAAGACGGCCGTACCCTC	CGCTGAACCTTTCAAGATCCATCG
EMB1345	AT2G26060	GGGATGGTCAAGATTTGGCA	CAAACCAACAGCAGTCACGGT
CB5-E	AT5G53560	TGAAGAAGTTTCAAAGCACAACA	TGAAGAAGTTTCAAAGCACAACA
RHIP1	AT4G26410	GAGCTGAAGTGGCTTCCATGAC	GGTCCGACATACCCATGATCC
TIP41	AT4G34270	GTGAAAACTGTTGGAGAGAAGCAA	TCAACTGGATACCCTTTCGCA

Supplementary Figure S1

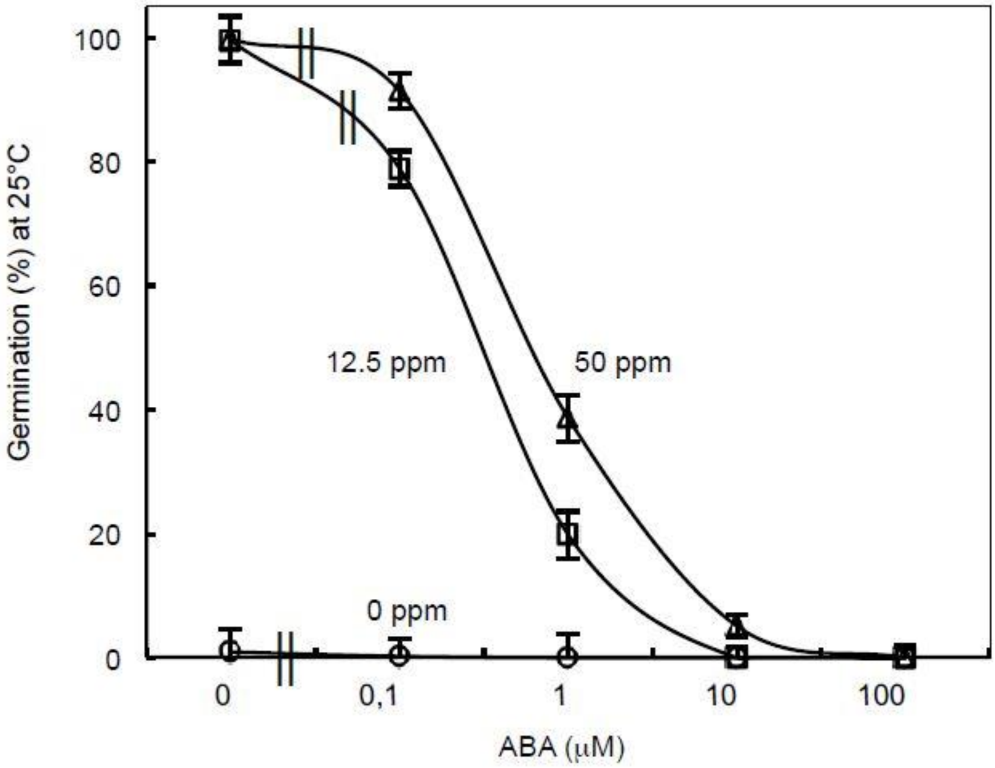

Figure S1 . Effects of ABA concentra0on on the germina0on percentage. obtained at 25°C with dormant Col seeds placed in the presence of 12.5 and 50 ppm ethylene. Dormant seeds did not germinate in absence of ethylene (0 ppm). Means of 3 replicates ± SD. Seeds harvested in 2015.
